# Atorvastatin combined with dexamethasone promote hematoma absorption in an optimized rat model of chronic subdural hematoma

**DOI:** 10.18632/aging.203717

**Published:** 2021-11-23

**Authors:** Dong Wang, Yueshan Fan, Jun Ma, Chuang Gao, Xuanhui Liu, Zilong Zhao, Huijie Wei, Guili Yang, Jinhao Huang, Rongcai Jiang, Jianning Zhang

**Affiliations:** 1 Department of Neurosurgery, Tianjin Medical University General Hospital, Tianjin 300052, Tianjin, China; 2 Tianjin Neurological Institute, Key Laboratory of Post-Neuroinjury Neurorepair and Regeneration in Central Nervous System, Ministry of Education and Tianjin City, Tianjin 300052, Tianjin, China; 3 Tianjin Medical University, Tianjin 300070, Tianjin, China

**Keywords:** atorvastatin combined with low-dose dexamethasone treatment, CSDH, optimized CSDH rat model, inflammatory activities, angiogenic processes

## Abstract

Previous studies found that atorvastatin and dexamethasone were effective in promoting the absorption of chronic subdural hematoma. In this study, we aimed to investigate the effect of pharmacotherapy in an optimized rat model of chronic subdural hematoma.

Rat model of chronic subdural hematoma via a bEnd.3 cell and Matrigel mix was established and dynamic changes in different drug treatment groups were tested. The hematoma gradually increased, peaked on the fifth day (263.8±52.85 μl), and was completely absorbed in two weeks. Notably, Kruppelle-like factor 2 expression was significantly decreased with increasing hematoma volume, and then increased in the repair period. The expression of IL-10 was increased and peaked on 7 days, and then decreased at 14 days. The dynamic trends of IL-6, IL-8, MMP-9, and VEGF were also increased first and then decreased. Both monotherapy and the combined treatment by atorvastatin and dexamethasone could counteract the inflammatory activities, decrease hematoma permeability, and improve hematoma absorption, however, most prominent in combined group. The combined treatment could more effectively increase Kruppelle-like factor 2 and ZO-1 expression, attenuate the expression of NF-κb. Most importantly, the combined treatment enhanced the neural functional prognosis and reduced the mortality of chronic subdural hematoma rats.

According to our results, the combined treatment could more effectively attenuate inflammatory. And it could also enhance angiogenic activities which could promote the stability of local function and structure of the hematoma cavity, reduce the hematoma volume and improve the outcomes of rats with chronic subdural hematoma than single treatments in the optimized chronic subdural hematoma model.

## INTRODUCTION

Chronic subdural hematoma (CSDH) is a common disease that occurs in elderly individuals. The main treatment for this disease is burr hole craniotomy [1]. Neurosurgery is successful in nearly 80% of patients, but 20% of patients still experience recurrence and require further surgery [2]. Because of the increasing elderly population and the use of anticoagulants, an increasing number of people suffer from these conditions [3]. Thus, it is necessary to identify nonsurgical ways as adjuvant therapy or another treatment option to rescue CSDH patients.

In our previous randomized controlled clinical trial, we demonstrated that the application of a low dose and a long course of atorvastatin can accelerate the absorption of CSDH and reduce the rate of operation. However, a poor effect was observed in 11.2% of the patients (11 operations/98 total) who were only treated with atorvastatin. Then, we applied atorvastatin combined with low-dose dexamethasone to treat patients with CSDH; this combination was much more effective than atorvastatin alone in decreasing the volume of hematoma in preliminary small-scale clinical observation studies [4, 5]. In previous *in vitro* studies, we also found that the combined treatment could counteract hematoma-induced injury in endothelial cells via changing the expression of Kruppelle-like factor 2 (KLF-2) [6, 7]. However, the mechanism of the combined treatment *in vivo* remains unclear and requires further study.

In our early subdural hematoma (SDH) studies, the function of capsule neovascularization was closely related to the absorption of the hematoma, and low-dose atorvastatin could promote the maturation of capsule neovascularization, attenuate the local abnormal inflammatory response, and accelerate the absorption of the hematoma [8, 9]. However, these acute blood injection models could partly mimic the process of hematoma injury, but not spontaneous formation or progressive enlargement which is found in the clinical patients. Based on these conditions, we optimized the model with a Matrigel and bEnd.3 cell mixture, which was characterized by spontaneous formation, the formation of a capsule and gradual enlargement of the hematoma [10]. Following this model, we aimed to further detect the mechanisms of the drug treatment effects on the dynamic changes of the hematoma.

We hypothesize that atorvastatin combined with low-dose dexamethasone could much more effectively counteract the abnormal inflammatory and pathological angiogenic processes than single treatment in the optimized CSDH model.

## RESULTS

### Dynamic changes in the hematoma volume in the optimized CSDH model

**Figure 1:**
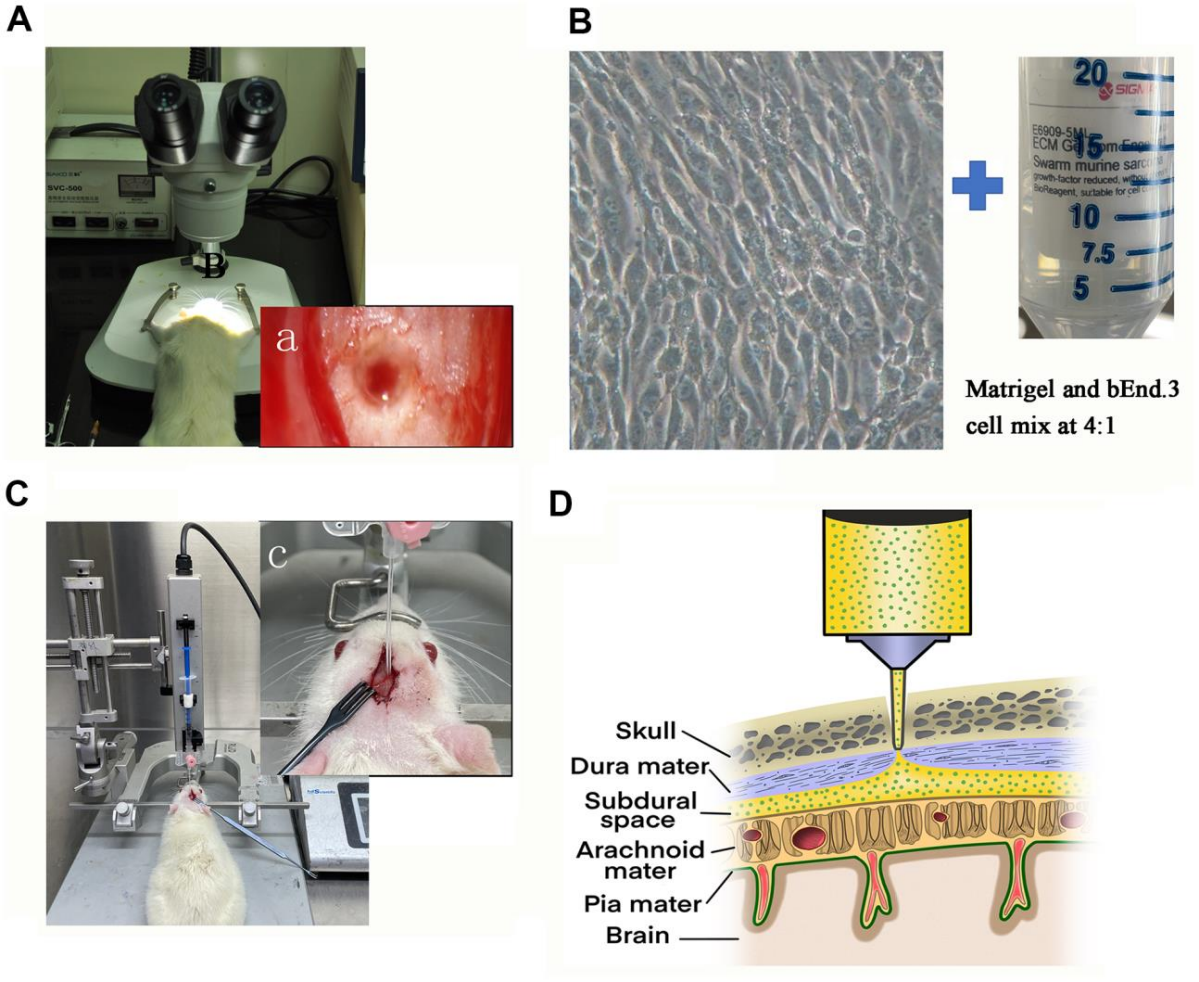
**Illustration of the rat model of CSDH.** (**A**) The sphenoid burr hole was made using a modified drill, and the dura was punctured under a microscope. (a) The microscopic photograph of the burr hole. (**B**) Prepare the cell-matrix glue mixture for injection. Representative photomicrograph of the morphology of bEnd.3 cells under light microscope and Sigma-Aldrich Matrigel. (**C**) A photograph of the entire assembly comprised of a stereotaxic frame and a stereotaxic injector with a 20-gauge catheter. (c) Zooming diagram of model rat. (**D**) Schematic diagram of drilled sphenoid burr hole, punctured dural hole, and catheter perfusion.

The design of the experiment was presented in Figure 1. We punctured the dura under a microscope (Figure 1A). The cell-matrix glue mixture was injected in the subdural space (Figure 1B–1D).

**Figure 2:**
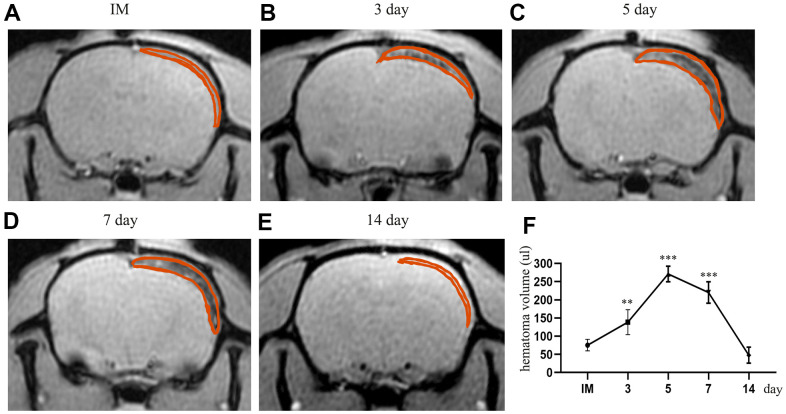
**The dynamic change in hematoma volume in the chronic subdural hematoma model.** (**A**) The MRI (GRE sequence) in the IM group. The part circled in red is the mixture of Matrigel and cells located in the subdural cavity of the rat; at this time, there is no blood signal in the subdural cavity. (**B**–**E**) Dynamic MRI (GRE) on the 3rd, 5th, 7th and 14th days after the establishment of the model. The part circled in red is the hematoma formed by spontaneous hemorrhage in the subdural region, in which the thickness and volume of the hematoma peaked at 5 d post-injury. At 14 d, most of the hematoma had been absorbed. (**F**) The dynamic change in the hematoma volume of the rats after modeling. IM: immediately after modeling. ** indicates p < 0.01 compared with the IM group, and *** indicates p < 0.001 compared with the IM group. IM indicates immediate MRI images after the establishment of the model.

We tested the volume of the hematoma immediately, 3 d, 5 d, 7 d, and 14 d by MRI spectrometry (GRE) after CSDH injury. We observed dynamic trends in the hematoma volume at different timepoints after modeling. The volume of the hematoma increased and peaked at 5d post-injury (263.8±52.85 μl, p<0.05). After the peak time, the hematoma was gradually absorbed (Figure 2A–2E). At 14 d, most of the hematoma had been absorbed (47.63±21.96 μl, p>0.05). Based on these results we demonstrated that this model successfully mimicked the chronic process of CSDH rather than the acute process.

### Expression changes in inflammatory and angiogenic factors in the capsule of the hematoma at different timepoints post-injury

**Figure 3:**
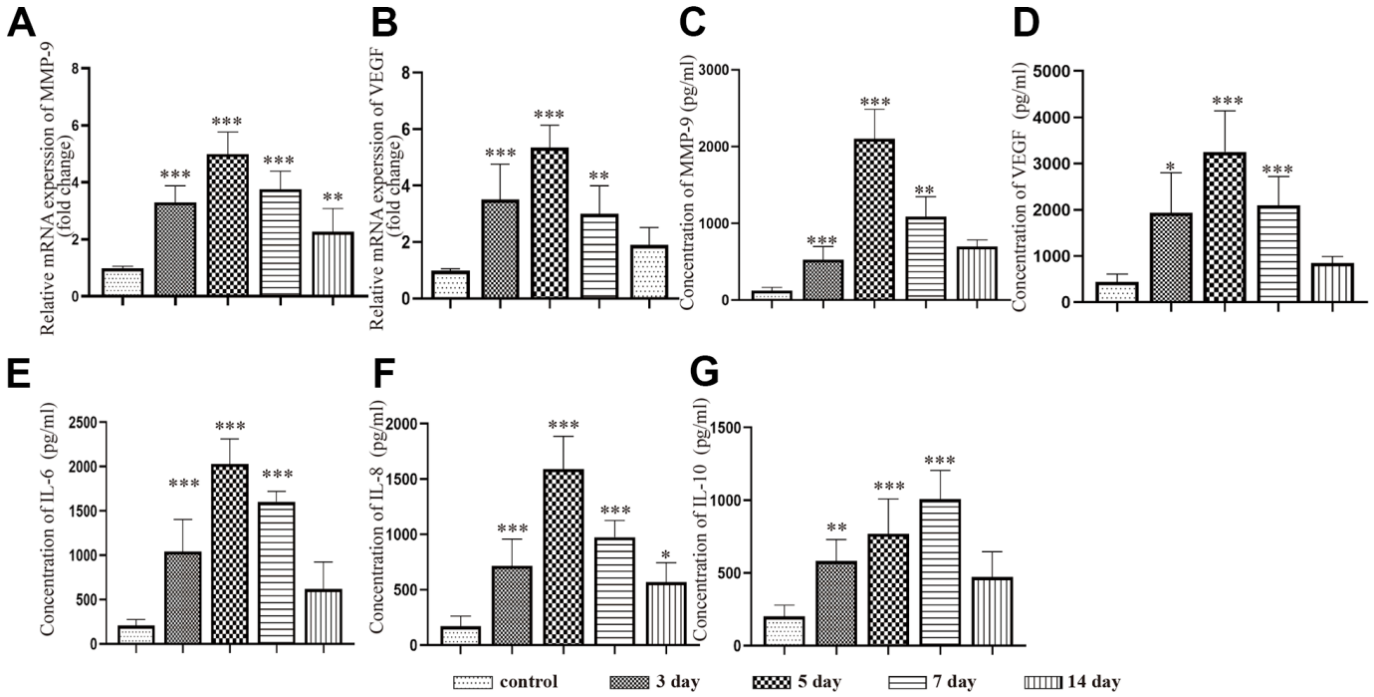
**Dynamic changes in the expression of inflammatory factors and angiogenic factors in the capsule of rats with chronic subdural hematoma after modeling.** (**A**) Changes in MMP-9 mRNA expression in the hematoma capsule at different timepoints after modeling. (**B**) Changes in VEGF mRNA expression in the hematoma capsule at different timepoints after modeling. (**C**) Changes in MMP-9 protein expression in the hematoma capsule detected by ELISAs at different timepoints after modeling. (**D**) Changes in VEGF protein expression in the hematoma capsule detected by ELISAs at different timepoints after modeling. (**E**) Changes in IL-6 protein expression in the hematoma capsule detected by ELISAs at different timepoints after modeling. (**F**) Changes in IL-8 protein expression in the hematoma capsule detected by ELISAs at different timepoints after modeling. (**G**) Changes in IL-10 protein expression in the hematoma capsule detected by ELISAs at different timepoints after modeling. IM: immediately after modeling. * p < 0.05 compared with the control group, ** p < 0.01 compared with the control group, *** p < 0.001 compared with the control group.

We performed PCR and ELISAs to test the changes in inflammatory and angiogenic factor expression in the neomembrane and capsule of the hematoma at different timepoints after CSDH injury. The mRNA expression of MMP-9 and VEGF was increased and peaked at 5 d. The expression of these mRNAs was decreased but still higher than that of the control (Figure 3A, 3B), which was consistent with the trend we detected by ELISAs. We also used ELISAs to test the levels of IL-6, IL-8 and IL-10 in the capsule of the hematoma. Similar trends in the expression of the MMP-9 and VEGF proteins were found (Figure 3C–3F). The expression of IL-10 was significantly increased and peaked at 7 d. After the peak time, the expression of IL-10 was reduced at 14 d post-injury but remained higher than that of the control (Figure 3G).

### KLF-2 expression after CSDH injury

**Figure 4:**
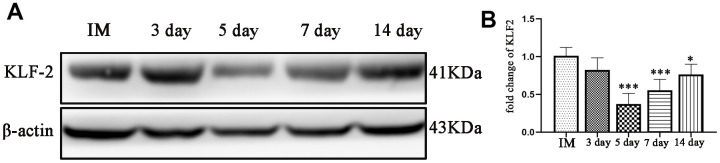
**Changes in KLF-2 expression in the capsule of the chronic subdural hematoma model.** (**A**) Changes in KLF-2 in the capsule of the hematoma at different timepoints after modeling. (**B**) The KLF-2 mRNA expression in the capsule of the hematoma detected by PCR at different timepoints after modeling. IM: immediately after modeling. * p < 0.05 compared with the control group, *** p < 0.001 compared with the IM group.

Western blotting and PCR were performed to detect the expression of KLF-2 in the neomembrane and capsule of the hematoma at different timepoints post-injury. The results showed that KLF-2 expression was inhibited with increasing hematoma volume and peaked at 5 d, reaching nearly half of the control expression, and then gradually increased at 7 d and 14 d but was not higher than that of the control group. More significantly, the trend of KLF-2 was opposite to that of the factors we tested previously (Figure 4A, 4B).

### The combined treatment could much more effectively reduce the hematoma volume after CSDH formation

Based on these data, we found the greatest hematoma volume and the largest changes in related protein expression at 5 d post-injury. We chose this timepoint for the following study.

**Figure 5:**
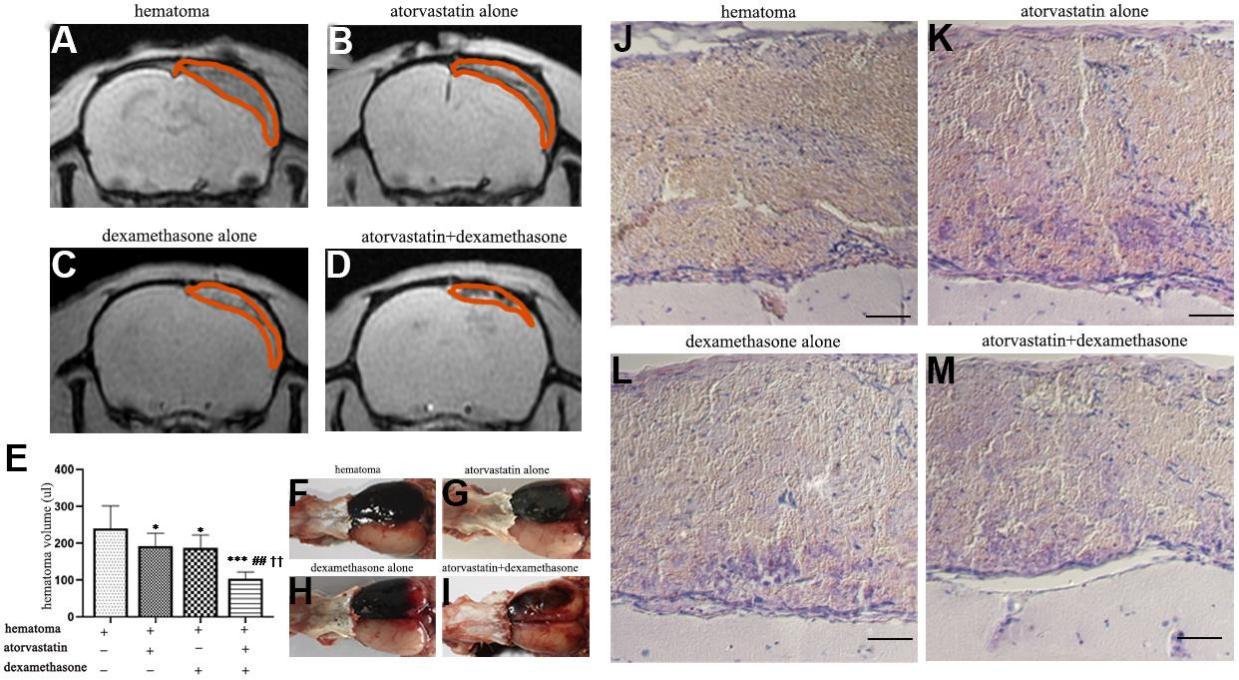
**MRI and HE staining showed hematoma volume changes in different intervention groups on the 5th day after modeling.** (**A**) Representative MRI (GRE) images of hematomas on the 5th day in the hematoma group. (**B**, **C**) Representative MRI (GRE) images of hematomas on the 5th day after treatment with atorvastatin and dexamethasone separately. (**D**) Representative MRI (GRE) images of hematomas on the 5th day in the atorvastatin + dexamethasone treatment group. (**E**) The changes in hematoma volume of the rats with CSDH after different treatments. (**F**–**I**) Typical images of growth appearances in different treatment groups after hematoma injury. (**J**–**M**) Representative H&E staining in different treatment groups after hematoma injury, bar =100 μm. * p < 0.05 compared with the control group, *** p < 0.001 compared with the control group, ## p<0.01 compared with the atorvastatin treatment group; †† p<0.01 compared with the dexamethasone treatment group.

MRI spectrometry (GRE) was performed to detect the changes in hematoma volume and histopathological characterization in different treatment groups post-injury (Figure 5A–5D). We further calculated the total hematoma volume at 5 d in different treatment groups (Figure 5E). The hematoma volume decreased to different degrees in both the monotherapy and combined treatment groups compared with the control group. Moreover, the results showed that in the combined treatment group, the hematoma volume was decreased by nearly half compared with that in the injury group. We observed large and thick hematoma neomembranes in the hematoma group. Robust fresh red blood cells and inflammatory cells infiltrated the hematoma cavity (Figure 5F–5M). Compared with that in the hematoma group, the hematoma cavity in the single atorvastatin and dexamethasone treatment groups had a smaller hematoma volume and lower inflammatory phagocytosis. Notably, the hematoma volume was significantly reduced, the number of fresh red blood cells in the hematoma cavity decreased, and the hematoma capsule was obviously thickened in the combined treatment group. These results suggested that compared with the single treatment, the combined treatment strongly improved the absorption of the hematoma at 5 d post-injury.

### The combined treatment decreased vascular permeability significantly after CSDH formation

**Figure 6:**
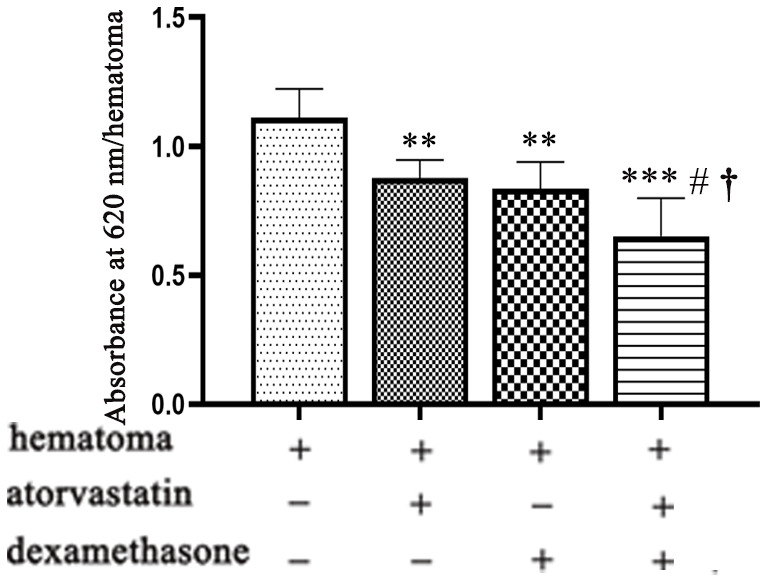
**Vascular permeability of the hematoma cavity in different intervention groups on the 5th day after modeling.** Quantification of Evans blue in the different treatment groups after CSDH modeling. ** means p < 0.01 compared with the control group; *** means p < 0.001 compared with the control group; # p<0.05, compared with the single atorvastatin treatment group; † p<0.05, compared with the single dexamethasone treatment group.

We detected high permeability in the capsule of the hematoma due to vascular leakage. Therefore, we measured vascular permeability by the evans blue test after establishing the CSDH model and administering different treatments (Figure 6).

We found that the combination treatment reduced the evans blue concentration in the hematoma cavity (Figure 6), suggesting that the leakage of new blood vessels was effectively reduced in the capsule of the hematoma.

### The combined treatment more effectively regulated the expression of inflammatory factors and angiogenic factors after CSDH formation

**Figure 7:**
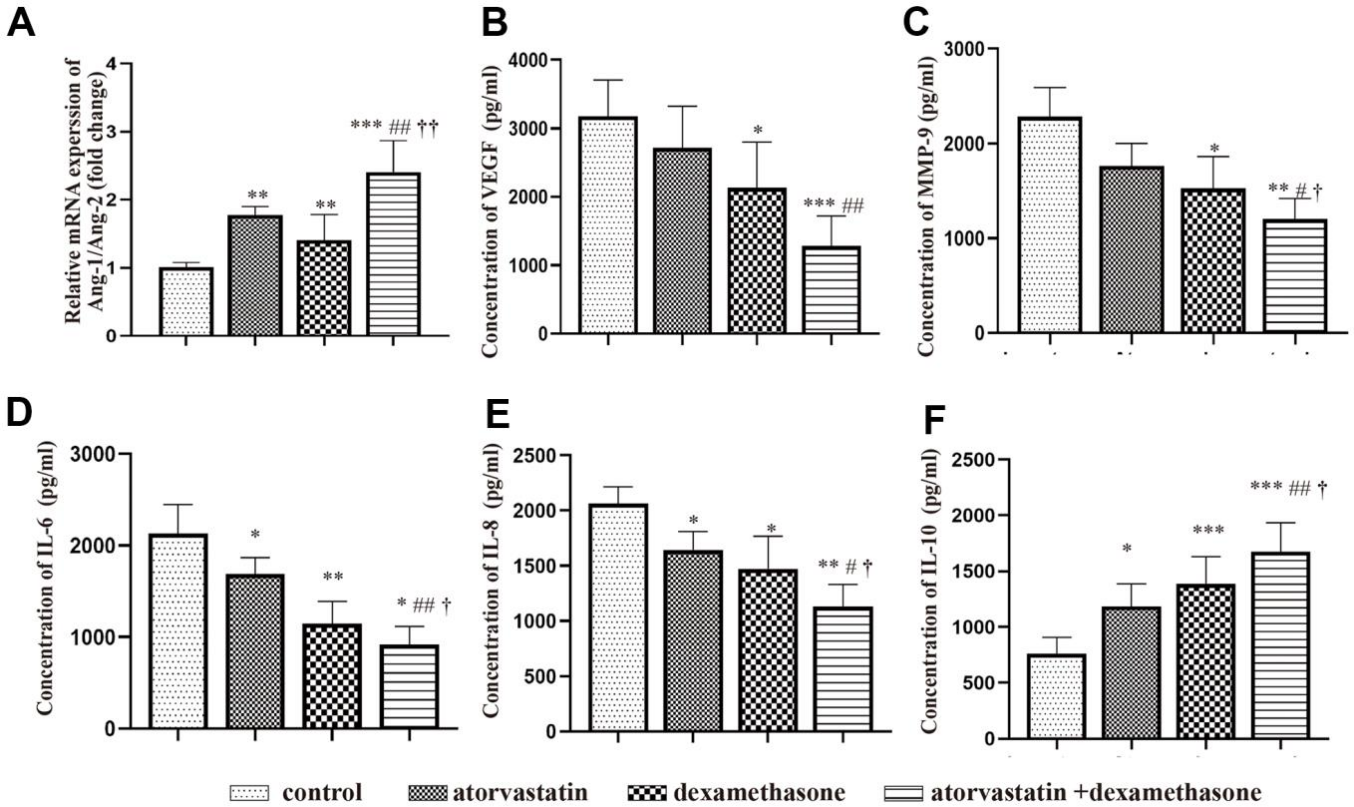
**Changes in the expression of inflammatory factors and angiogenic factors in different treatment groups after chronic subdural hematoma modeling.** (**A**) Changes in the Ang-1/Ang-2 mRNA ratio in the hematoma capsule of different treatment group after modeling. (**B**) Changes in VEGF protein expression in the hematoma capsule of different treatment group after modeling. (**C**) Changes in MMP-9 protein expression in the hematoma capsule detected by ELISAs of different treatment group after modeling. (**D**) Changes in IL-6 protein expression in the hematoma capsule detected by ELISAs of different treatment group after modeling. (**E**) Changes in IL-8 protein expression in the hematoma capsule detected by ELISAs of different treatment group after modeling. (**F**) Changes in IL-10 protein expression in the hematoma capsule detected by ELISAs of different treatment group after modeling. * p < 0.05 compared with the control group, ** p < 0.01 compared with the control group, *** p < 0.001 compared with the control group; # p<0.05, compared with the single atorvastatin treatment group, ## p<0.01, compared with the single atorvastatin treatment group; ^†^ p<0.05, compared with the single dexamethasone treatment group; ^††^ p<0.01, compared with the single dexamethasone treatment group.

We performed PCR and ELISAs to test the changes in inflammatory factor and angiogenesis factor expression in the neomembranes of hematomas in different treatment groups post-injury. The results suggested that the ratio of Ang-1 and Ang-2 mRNA expression was increased in all treatment groups, indicating more robust mature vessels in the neomembrane (Figure 7A). Besides, we performed ELISAs to evaluate the expression of MMP-9 and VEGF in the hematoma capsules. We found that the levels of both MMP-9 and VEGF were decreased after treatment. Notably, this effect was more obvious in the combined treatment group than in the other groups (Figure 7B, 7C). Then, the levels of IL-6, IL-8 and IL-10 in the capsule of the hematoma were further measured to evaluate the inflammatory response. The MMP-9 and VEGF proteins showed similar trends, which were also consistent with those of IL-6 and IL-8 (Figure 7D, 7E). However, the expression of IL-10 was significantly increased after treatment of the injured rats (Figure 7F).

### The combined treatment can remarkably attenuate local inflammation and endothelial injury

**Figure 8:**
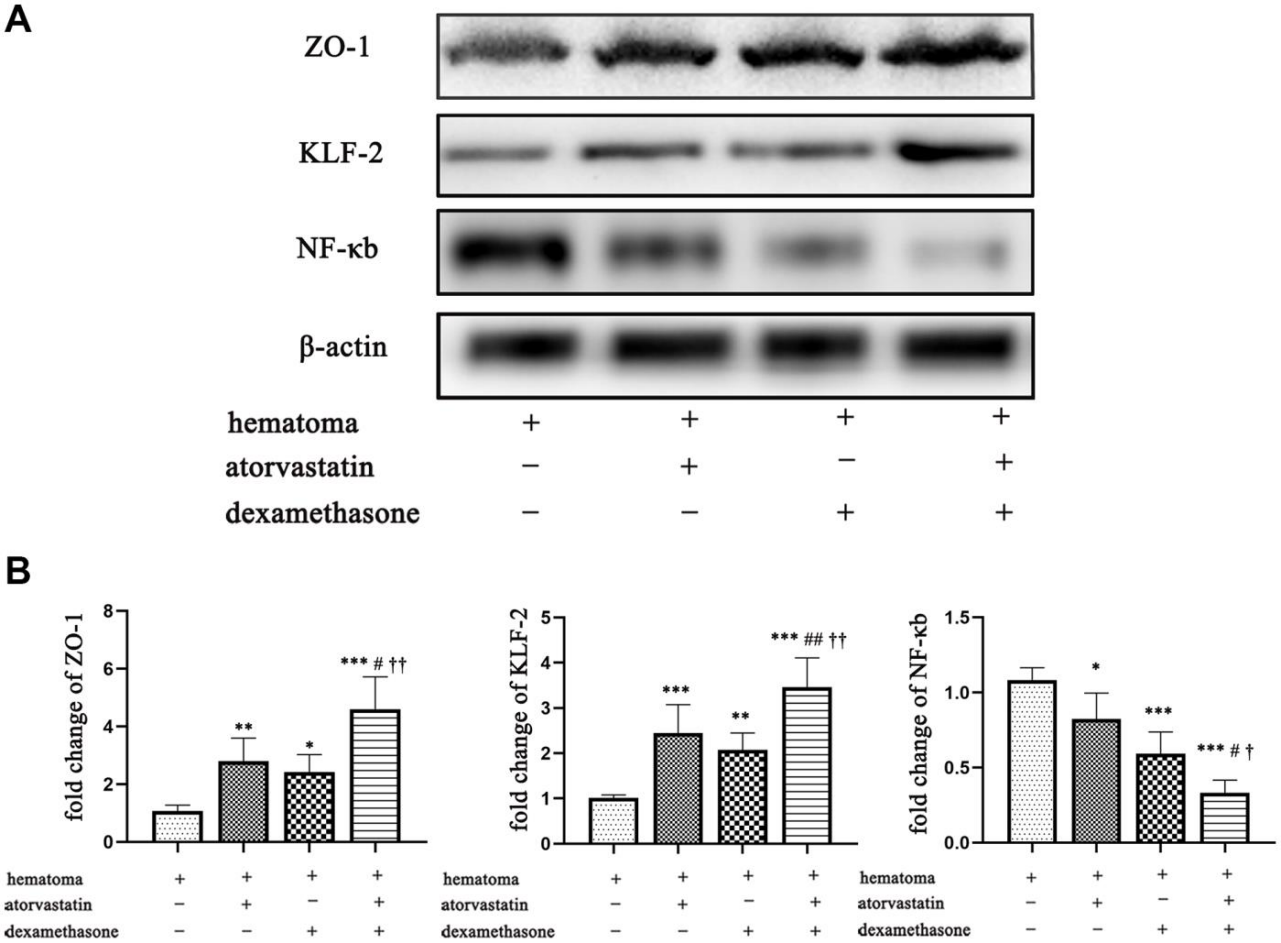
**Changes in ZO-1, KLF-2 and NF-κb expression in the capsule of the chronic subdural hematoma model in different treatment groups.** (**A**) Changes in ZO-1, KLF-2 and NF-κb expression in the hematoma capsule in the different treatment groups after modeling. (**B**) The ZO-1, KLF-2 and NF-κb mRNA expression in the hematoma capsule detected by PCR in the different treatment groups after modeling. * p < 0.05 compared with the control group, *** p < 0.001 compared with the hematoma group. # indicates p<0.05, compared with the atorvastatin-only treatment group, ## indicates p<0.01, compared with the atorvastatin-only treatment group; † indicates p<0.05, compared with the dexamethasone-only treatment group; †† indicates p<0.01, compared with the dexamethasone-only treatment group.

Then, we detected the expression of NF-κb and ZO-1 in the different treatment groups post-injury by Western blots and PCR analyses. We found that the combined treatment attenuated the expression of NF-κb and enhanced the expression of KLF-2 and ZO-1 compared with the single treatments (Figure 8A), resulting in reducing the leakage of new blood vessels in the capsule of the hematoma. The same phenomenon was also shown in the PCR assay (Figure 8B).

### The combined treatment significantly improved the neurological functional outcome after CSDH injury

**Figure 9:**
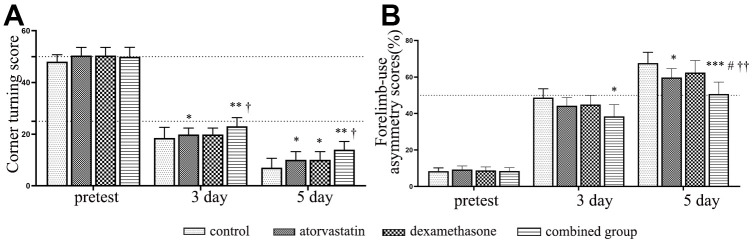
**Dynamic changes in the functional scores of the rats after different interventions.** (**A**) The dynamic changes in corner turning test scores in different intervention groups. (**B**) The dynamic changes in forelimb-use asymmetry scores in different intervention groups. *Compared with the hematoma group, p < 0.05; ** compared with the hematoma group, p < 0.01; *** compared with the hematoma group, p < 0.001; # compared with the atorvastatin treatment group, p < 0.05; † indicates p<0.05, compared with the dexamethasone-only treatment group; †† indicates p<0.01, compared with the dexamethasone-only treatment group.

We further evaluated the neurological functional outcome by corner turning test scores and forelimb-use asymmetry scores in different treatment groups at different timepoints post-injury. Firstly, we calculated the number of rats died during the experiment timepoint in the different treatment group. We found that a total of 10 (36%) rats died in the CSDH group, which was much higher than the monotherapy group and combined treatment (atorvastatin group: n=6 (33%); dexamethasone group: n=4 (28%); combined group: n=3 (17%)). As shown in Figure 9, the rats in the hematoma group exhibited lower scores at all timepoints post-injury for the corner turning test scores, which indicated poor outcomes, than the rats in the treatment groups. In the forelimb-use asymmetry score, higher scores indicate more serious neurological dysfunction. The scores were strongly increased at 3 d and 5 d. However, the result indicated that the scores decreased in the treatment group, especially in the combined treatment group, compared with the injury group at 3 d and 5 d. All of the results indicated that the rats with CSDH have severe neurological functional deficits, but the combined treatment could more effectively rescue the injured rats and improve their neurological outcomes than the single treatments.

## DISCUSSION

In this study, we found that a processive subdural hematoma formed with an increasing hematoma volume, which peaked at 5 d (Figure 2). Besides, with the changes in hematoma volume, there were concomitant trends in the expression of proinflammatory and proangiogenic factors, which peaked at 5 d and then gradually decreased. Furthermore, the expression of KLF-2 was inhibited with the gradually increasing hematoma volume, and then gradually increased at 7 d and 14 d, indicating a correlation between the expression of KLF-2 and the progression of the disease. In this optimized model, we found the combined treatment effectively increased the expression of KLF-2 and ZO-1, attenuated the expression of NF-kB, which improved the inflammatory reaction, stabilized the neovascularization, and rapidly decreased the hematoma volume, resulting in the lowest mortality when comparing to other treatment groups. These data show the optimized model may be similar with CSDH patients in the same pathophysiological process. In addition, the combined treatment notably improved the neurological function outcomes of the rat model of CSDH.

In recent years, an increasing number of studies have demonstrated that angiogenesis plays pivotal roles in the process of CSDH [11–13]. In addition, some studies have reported that the ratio of Ang-1 to Ang-2 plays important role in unstable neovessel formation, causing high vascular leakage in patients with CSDH [12]. Moreover, the ratio of Ang-1/2 was most significantly regulated by VEGF. In patients with CSDH, the concentration of VEGF in the hematoma is very high, and VEGF plays a critical role in this disease, which is essential for neovessel formation and remodeling [14]. In addition, there were higher concentrations of IL-6, IL-8, and IL-10 in the hematoma fluid than in the serum [15]. Based on a previous study by our research group, the abnormal function of new blood vessels in the hematoma wall and the gradual increase in the hematoma caused by blood leakage were considered, and the animal model was optimized. The model was established by using the characteristics of bEnd.3 cells that secrete high levels of VEGF, resulting in abnormal neovascularization and abnormal function. We generated a rat model of CSDH by injecting a bEnd.3 cell-Matrigel mixture into the subdural space to mimic the pathological environment of abnormal inflammatory and pathological angiogenic processes which were found in clinical patients, characterized by a spontaneously and gradually enlarged hematoma volume, this process is completely different to the traditional method. Thus, we found that abnormal inflammation and neovascularization in the hematoma cavity are the main causes of neovascularization dysfunction, increasing permeability and promoting blood leakage and gradual enlargement of hematoma, which are also consistent with our previous conclusions [5, 6, 16]. Based on these results, our model may become a more convincing tool in further study in the field of the mechanism of CSDH and develop new therapies.

Robust studies have reported that atorvastatin plays important roles in angiogenesis and anti-inflammatory effects [9, 17–19], which are closely linked with the pathogenic process of CSDH. Similarly, early evidence indicated that corticosteroids could inhibit inflammation in chronic inflammatory disease [20], which has also been demonstrated in CSDH [21, 22]. However, in a trial of dexamethasone for CSDH, treated with high dose dexamethasone resulted in fewer better outcomes, and with high adverse events than placebo group at 6 months, which has published in The New England Journal of Medicine [23]. The study of dexamethasone in the treatment of chronic subdural hematoma shows that the curative effect of dexamethasone on chronic subdural hematoma is still controversial, which needs further study. Notably, in our previous preliminary proof of concept (POC) clinical trial, the results indicated that atorvastatin combined with low-dose dexamethasone effectively decreased the hematoma volume of patients compared with atorvastatin alone [5]. In addition, in our *in vitro* studies, we found that atorvastatin combined with low-dose dexamethasone could protect endothelial cells from hematoma injury and that KLF-2 played pivotal roles in the effects of the combined therapy [6, 7]. In this research, we obtained similar results in animal models and promising findings on the repair of nerve function damage (Figure 9).

KLF-2 expression was strongly related to the hematoma volume; it was inhibited with increasing hematoma volume and then gradually increased in the healing period. In addition, this result is similar with our findings in a previous *in vitro* study. Combined with these studies, our results indicated that KLF-2 plays pivotal repair roles in our rat model of CSDH. Most importantly, no reports have examined the drug’s effects on animal models of CSDH. KLF-2 plays an important role in vascular function as a member of the zinc-finger transcription factors [24, 25]. Robust studies have convincingly shown that KLF-2 is highly expressed in endothelial cells and plays a critical role in regulating vessel function [25, 26]. In addition, some studies showed that the decreased expression of KLF-2 was closely associated with changes in vascular permeability [27].

Recent evidence has shown that KLF-2 is not limited to its role in regulating vascular function but also plays key roles in inflammatory conditions [28, 29]. Hence, the role of KLF-2 in NF-κb-mediated inflammatory activities and inflammatory diseases has been investigated and identified in many studies [30, 31]. The results showed that the level of KLF-2 was gradually reduced after CSDH injury and then increased at 7 d and 14 d, but this trend was reversed for the proangiogenic and proinflammatory factors we tested after the CSDH model. In addition, these findings contrasted with the trends of hematoma volume but were consistent with the model rat outcomes. Then, we found that the combined treatment could strongly attenuate the expression of NF-κb and enhance the expression of KLF-2, with the more effective hematoma absorption and a better outcome, compared with the monotreatment’s in the CSDH rat model at 5 d. This result was also consistent with previous studies, indicating that NF-κb participated in the development of CSDH and the combined treatment could reduce the inflammatory activities in the neomembrane via the NF-κb pathway [32, 33]. Thus, we speculated that KLF-2 plays pivotal repair roles during hematoma formation.

We found that the hematoma volume peaked at 5 d with obvious changes in related protein expression. We chose this timepoint for the following study. We found that the expression of VEGF was decreased in the combined treatment group after modeling with an increased ratio of Ang-1 to Ang-2 (Figure 7), which indicated that the number of mature and stable blood vessels was increased, causing hematoma absorption and reducing vascular leakage [34, 35]. In addition, the combined treatment effectively decreased hematoma volume and vascular leakage (Figures 5, 6) and was related to reduced proinflammatory factor (IL-6, IL-8) and angiogenic mediator (MMP-9) expression at 5 d (Figure 7); these factors are necessary for neovessel maturation and reduced vascular inflammation [12, 15, 36, 37].

However, there are some limitations in our studies. We used normal rats but not CSDH-susceptible rats to optimize the CSDH model. In addition, bEnd.3 cells could not survive and sustain high levels of vascular injury factors in the subdural space for a long time. This phenomenon will cause absorption of the hematoma due to the strong repairability of the rats. Therefore, animal experiments cannot completely mimic the process of clinical patients with CSDH. Our study only examined one of the possible factors of CSDH formation. We only demonstrated that KLF-2 participated in the progression of CSDH. However, there are still no clear mechanisms between KLF-2 and the changes in related inflammatory vascular indexes and hematoma volume in the CSDH animal model, which need to be verified by further experiments.

## CONCLUSIONS

In conclusion, we demonstrated that atorvastatin combined with low-dose dexamethasone treatment could much more effectively improve inflammatory reactions and stabilize neovascularization, reduce the volume of hematoma and improve the outcomes of CSDH rats than single treatments. The association between KLF-2 and ZO-1 or NF-κb pathways may play pivotal roles in the process of hematoma formation and the prognosis of CSDH.

## MATERIALS AND METHODS

### Animals

We obtained adult Sprague Dawley (SD) rats (350-400g,) from Beijing HFK Bioscience (Beijing, China). The rats were housed in the facilities of Tianjin Medical General Hospital. All procedures were approved by the Ethics Committee of Tianjin Medical University General Hospital (Tianjin, China).

### Cell culture and CSDH model

BEnd.3 cells were purchased from the ATCC (ATCC, Manassas, VA, USA) and cultured in Dulbecco’s modified Eagle’s medium (DMEM, Corning, Tewksbury, MA, USA) with 10% fetal bovine serum (FBS, 26140079, Gibco, Waltham, MA, USA). We changed the medium every other day.

We performed the CSDH model as our previous study [10]. Matrigel E6909 (Sigma-Aldrich, St. Louis, MO, USA) was placed at 4° C overnight (the colloid liquid state was maintained) before use. We suspended the cells in sterile PBS with a concentration of 4 × 10^6^ cells/ml, and then mixed them with Matrigel at a ratio of 4:1. The final concentration of the cell-Matrigel mix was 10^6^ cells/ml. We placed the mix on ice to maintain the Matrigel liquid state before use.

We generally anesthetized the rats by intraperitoneal injection of 10% chloral hydrate (3 ml/kg). Then, the anesthetized rats were placed in a stereotaxic frame (RWD Life Science, Shenzhen, China). We made a sphenoid burr hole over the right coronal suture with dural integrity. Then, under a microscope (RWD Life Science), the dura was punctured by a 0.2 mm diameter needle. A tapered tip (20G, BD Venflon^TM^, Helsingborg, Sweden) was fitted tightly into the hole without contacting the dura. The details of the method are described in our previous studies [8, 36, 38]. A 150 μl mixture or Matrigel alone was injected into the subdural space by a stereotaxic injector (RWD Life Science) at a rate of 25 μl/min. We removed the needle in 10 min until the gel solidified after finishing the injection.

### Magnetic resonance imaging (MRI)

We used 3.0T MRI (MR750, GE Healthcare, Chicago, IL,USA) to identify whether the CSDH model was successfully established and to test the hematoma volume. Briefly, immediately and 3, 5, 7, and 14 d after surgery (n=30), anesthetized rats were positioned appropriately with the coil in the MR scanner. MRI images were acquired with the GRE sequence. We calculated the volume of the hematoma at different timepoints by the GE workstation.

### Experimental group

Rats with CSDH were randomly separated into four groups: the control group (saline, n=18), single atorvastatin group (3 mg/kg/day, Pfizer, USA, n=18), single dexamethasone group (0.405 mg/kg, Tianjin Pacific Pharmaceutical Co., Ltd., China; n=18), and atorvastatin and dexamethasone combined treatment group (atorvastatin:3 mg/kg/day, dexamethasone: 0.405 mg/kg; n=18). We treated the rats at 1d post-injury by gavage administration, and the period of the treatment was 5 d.

### Tissue preparation

In the model group, we sacrificed the rats immediately (n=6) and at 3 d (n=6), 5 d (n=6), 7 d (n=6), and 14 d (n=6) to test the expression of related proteins and mRNAs by enzyme-linked immunosorbent assays (ELISA) and quantitative real-time polymerase chain reaction (qRT-PCR). Besides, western blot was performed with 18 rats (immediately: n=6; 3 d: n=6; 5 d: n=6; 7 d: n=6; 14 d:n=6) to evaluate the expression of proteins. There were 72 rats in the treatment group at 5 d (control: n=18; atorvastatin: n=18; dexamethasone: n=18; combined group: n=18) for H&E staining, Evans blue analysis, ELISAs, and PCR. The tissues were collected and fixed with 4% paraformaldehyde (PFA). Thereafter, the capsules of hematoma in different groups were collected and stored at -80° C for Western blotting, ELISAs, and PCR analysis at 5 d. For Evans blue injection and detection, the rats were infected with dye (2%, 1 ml/kg) through the right femoral vein and circulated for 30 min, and then euthanized under anesthesia. The neomembrane of the hematoma was dissected and fixed with 4% PFA. For Evans blue analysis, the tissues were incubated with formamide (60° C, overnight). The absorbance values of the extract at 620 nm were measured.

### H&E staining

We performed as previous protocols [16]. At 5 days, the rats were killed and fixed with 4% paraformaldehyde. Brain tissue was collected and immediately frozen in - 80° C refrigerator for 10 minutes. Histologically, the part containing hematoma and dura was examined for 10 mm per 0.5 mm thickness.

### qRT-PCR

qRT-PCR was performed as described in our previously published protocol [16]. The primers we used were as follows:

MMP-9: forward 5′-TTCAAGGACGGTCGGTATT-3′

reverse 5′-CTCTGAGCCTAGACCCAACTTA-3′;

VEGF: forward 5′-GAGCAGGAGCCGAAGCC-3′

reverse 5′-GAGCCCAGAAGTTGGACGA-3′;

KLF-2: forward 5′-TGCCGTCCTTTGCCACTTTCG-3′

reverse 5′- GCACGCTGTTTAGGTCCTCATCC-3′;

NF-κb: forward 5′- TGTGGTGGAGGACTTGCTGAGG -3′

reverse 5′- AGTGCTGCCTTGCTGTTCTTGAG-3′;

ACTB: forward 5′-GAAGTACCCCATTGAACACGG-3′

reverse 5′-TGGGTCATCTTTTCACGGTTG-3′

ZO-1: forward 5′-ATAAAGAGAAAGGTGAAACACTGCT-3′

reverse 5′-TCACAGTGTGGTAAGCGCAG-3′

### ELISA and western blotting

We tested the expression of MMP-9, VEGF, IL-6, IL-8, and IL-10 by ELISA kits (R&D Systems, Minneapolis, MN, USA) according to the manufacturer’s instructions. We measured the absorbance at 450 nm via a plate reader (Molecular Device, Sunnyvale, CA, USA). Western blotting was performed as described in previous studies [39]. Anti-KLF-2 (1:1000, Abcam, ab17008), anti-NF-κb (1:1 000, Cell Signaling Technology, 8242s, Danvers, MA, USA) and anti-ZO-1 (1:1000, Cell Signaling Technology), were used as the primary antibodies. Thereafter, horseradish peroxidase (HRP)-conjugated secondary IgG was incubated with the membranes for 1 h at room temperature. An enhanced chemiluminescence (ECL) system (Millipore, Billerica, MA, USA) was used to expose the membranes. We tested the gray value by ImageJ software.

### Corner turning score and forelimb-use asymmetry score

The corner turning score and forelimb-use asymmetry score were calculated before induction of the CSDH model and repeated on days 3, 5 in the different treatment groups post-injury. We experimented with the steps in a previous study [8]. We used a blinded method to evaluate the experimental treatment.

### Statistical analysis

GraphPad Prism (IBM, USA) was used to analyze the results and they are presented as the mean ± SD. We performed one-way ANOVA to analyze data. P<0.05 was defined as significant.
